# 

**Published:** 1878-05

**Authors:** 


### BOOKS AND PAMPHLETS RECEIVED.

Cyclopædia of the Practice of Medicine. Edited by Dr. H. Von Ziemssen, Prof. of Clin. Med. Munich, Bavaria. Vol. XVII. General Anomalies of Nutrition and Poisons. By Prof. H. Immerman, of Basel; Prof. R. Boehm, of Dorpat; Prof. B. Naunyn, of Kœnigsberg; and Prof. H. Von Bœck, of Munich. Albert H. Buck, M. D. American editior. New York: Wm. Wood & Co. 1878. 8 vo., cloth; pp. 968.

Handbook of Ophthalmology. By Prof. C. Schweiger, of the University of Berlin. Translated from the third German edition. By Porter Farley, M. D., Rochester, N. Y. With diagrams and other illustrations. Philadelphia: J. B. Lippincott & Co. 1878. 8 vo., cloth; pp. 546. Price, $4.50

On the Source of Muscular Power. Arguments and Conclusions drawn. from Observations upon the Human Subject under Conditions of Rest, and of Muscular Exercise. By Austin Flint, jr., M. D. New York: D. Appleton & Co. 1878. 12 mo., cloth; pp. 103. Price, $1.00.

Montreal General Hospital; Pathological report for the year ending May 1st, 1877. By Wm. Osler, M. D., of McGill University. Vol. I. Montreal: Dawson Brothers. 1878. 8 vo., cloth; pp. 97.

The Vest-pocket Anatomist (founded upon Gray). By C. Henri Leonard, A. M., M. D. Multum in Parvo. 2nd enlarged edition, sixth thousand. Copyright, 1875–1878. Detroit: 1878. Price, 50 cts.

Transactions of Iowa and Illinois Central District Medical Association, at the Meeting held in Board of Education Rooms, April 11, 1878, Rock Island, Illinois.

The Therapeutical Society of New York. (Reprint, with additions from the *New York Med. Journal*, Feb., 1878.)

A Review of the Treatment of Fracture of the Femur. By Edward Birch, M. D. (Reprint from the *St. Louis Med. and Surg. Journal*, March, 1878.)

Medical Organizations and their Value. An address delivered before the Alumni Association of Jefferson Medical College, March 9, 1877. By William B. Atkinson, M. D. Published by order of the Association.

Annual Announcement of Lectures at Toland Hall, Medical Department of the University of California, San Francisco. California Session of 1878.

### ANNOUNCEMENTS FOR THE MONTH.

### SOCIETY MEETINGS.

Chicago Medical Society—Mondays, May 6 and 20.

Chicago Society of Physicians and Surgeons—Mondays, May 13 and 27.

### CLINICS.

MONDAY.

Eye and Ear Infirmary—2 to 4 p. m., by Prof. Holmes and Dr. Hotz—2 p. m., Prof. Jones.

Mercy Hospital—2 to 3 p. m. Surgical, by Prof. Andrews.

Rush Medical College—1:30 p. m. Medical, by Dr. Bridge; 2:30 p. m. Dermatological and Venereal, by Dr. Hyde.

County Hospital—8 p. m. Necropsy, by Dr. Danforth.

Woman's Medical College—3 p. m. Surgical, by Prof. Owens.

TUESDAY.

County Hospital—1:30 p. m. Medical, by Prof. Lyman; 2:30 p. m. Surgical, by Prof. Parkes.

Mercy Hospital—2 p. m. Medical, by Prof. Hollister.

Eye and Ear Infirmary—2 p. m. Prof. Jones.

WEDNESDAY.

County Hospital—1:30 p. m. Ophthalmological, by Dr. Montgomery. 2:30 p. m. Gynecological, by Dr. Bridge.

Mercy Hospital—2 p. m. Eye and Ear, by Prof. Jones.

Rush Medical College—4 p. m. Diseases of the Chest, by Dr. E. Fletcher Ingals.

THURSDAY.

Mercy Hospital—2 p. m. Medical, by Prof. Davis.

Rush Medical College—1:30 p. m. Neurological, by Prof. Lyman.

Eye and Ear Infirmary—2 to 4 p. m. Operations by Prof. Holmes and Dr. Hotz.

FRIDAY.

Mercy Hospital—2 p. m. Medical, by Prof. Davis.

County Hospital—1:30 p. m. Medical, by Prof. Quine; 2:30 p. m., Surgical, by Prof. Powell.

Woman's Medical College—10 p. m. Ophthalmological, by Dr. Montgomery.

SATURDAY.

Rush Medical College—2 p. m. Surgical, Prof. Gunn.

Chicago Medical College—2 p. m. Surgical, by Prof. Andrews and Isham; 3 p. m., Diseases of the Chest, by Prof. Johnson.

Woman's Medical College—12 m. Gynecological, by Prof. Fitch; 3 p. m. Dermatological, Dr. Maynard.

Special Clinics daily, from 2 to 4 p. m., at the South Side Dispensary, and at the Central Free Dispensary.

For schedule of lectures at the colleges, apply to the college janitors.

